# Gauze packing as damage control for uncontrollable haemorrhage in severe thoracic trauma

**DOI:** 10.1308/003588413X13511609956057

**Published:** 2013-01

**Authors:** Y Moriwaki, H Toyoda, N Harunari, M Iwashita, T Kosuge, S Arata, N Suzuki

**Affiliations:** Yokohama City University Medical Center,Japan

**Keywords:** Gauze packing, Damage control, Gauze infection, Abdominal trauma, Uncontrolled haemorrhagic shock

## Abstract

**Introduction:**

The usefulness of thoracic damage control (DC) for trauma requiring a thoracotomy is not established. The aim of this study was to clarify the usefulness of thoracic packing as DC surgery.

**Methods:**

This was a retrospective case series study of 12 patients with thoracic trauma suffering uncontrollable intrathoracic haemorrhage and shock who underwent intrathoracic packing. Our thoracic DC technique consisted of ligation and packing over the bleeding point or filling gauze in the bleeding spaces as well as packing for the thoracotomy wound. The success rates of intrathoracic haemostasis, changes in the circulation and the volume of discharge from the thoracic tubes were evaluated.

**Results:**

Packing was undertaken for the thoracic wall in five patients, for the lung in four patients, for the vertebrae in two patients and for the descending thoracic aorta in one patient. Haemostasis was achieved successfully in seven cases. Of these, the volume of discharge from the thoracic tube exceeded 400ml/hr within three hours after packing in three patients, decreased to less than 200ml/hr within seven hours in six patients and decreased to 100ml/hr within eight hours in six patients. Systolic pressure could be maintained over 70mmHg by seven hours after packing.

**Conclusions:**

Intrathoracic packing is useful for some patients, particularly in the space around the vertebrae, at the lung apex, and between the diaphragm and the thoracic wall. After packing, it is advisable to wait for three hours to see whether vital signs can be maintained and then to wait further to see if the discharge from the thoracic tube decreases to less than 200ml/hr within five hours.

Although most cases of intrathoracic haemorrhage can be easily controlled with non-operative management such as simple thoracic drainage using thoracic tubes, we often encounter patients not only with intrathoracic haemorrhage but also with massive and rapid extrathoracic haemorrhage (such as intra-abdominal haemorrhage, retroperitoneal haemorrhage and external haemorrhage), whose intrathoracic haemorrhage cannot be controlled easily. The continuation of active haemorrhage despite shock and treatment against this condition (eg bolus infusion and massive transfusion) induces worsening physiological parameters, specifically acidosis, hypothermia and coagulopathy (lethal triad). The altered thoracic drainage volume may be a useful parameter for identifying a tendency toward the lethal triad and uncontrollable bleeding. Alternatively, this parameter could serve as a signal for the need for massive amounts of precious blood products.

We usually implement aggressive strategies for damage control (DC) in these patients, consisting of immediate control of haemorrhage and contamination followed by definitive reconstruction during a planned reoperation. DC is generally performed in patients with severe abdominal trauma and massive intra-abdominal haemorrhage.^1–6^ However, the usefulness of the abbreviated initial operation in DC for thoracic trauma is not established.^7–12^ It is not clear for what type of thoracic injury DC is effective or, indeed, which DC procedure is effective for thoracic surgery. We report a case series that extends the spectrum of the DC approach to the thorax to outside the abdomen. The objective of this study was to clarify the usefulness of gauze packing for haemostasis as DC surgery in patients with thoracic trauma.

## Methods

Medical records for the previous 8 years were reviewed for 12 patients suffering from chest trauma with uncontrollable intrathoracic haemorrhage who underwent intrathoracic packing as DC surgery. The changes in circulatory condition and the volume of discharge from the thoracic tube were evaluated. We have no clear mechanical or physiological indications for packing but in this study the indications for DC including gauze packing for thoracic trauma were:

> haemorrhagic shock owing to intrathoracic bleeding, which required an emergency thoracotomy;
> severe acidosis in arterial blood gas analysis, macroscopic coagulopathy and hypothermia in operative findings during thoracotomy (lethal triad); and
> no other way to stop the bleeding in a short time.

Our DC technique for thoracic trauma consisted of:

1. ligation of the bleeding point (eg thick vessels of the lung or pulmonary parenchyma) but without ligation of bleeding points on the thoracic wall;
2. gauze packing over the ligating point or bleeding point of the thoracic wall, with unfolded, thick, large gauze on the bleeding points or filling gauze in the bleeding spaces; and
3. gauze packing for the thoracotomy wound or suturing of the thoracotomy wound in association with the use of packing gauze.

When closing the thoracotomy wound, gauze was placed between the bilateral edges of the wound and the dissected spaces between the thoracic wall muscles, and both edges of the wound were pulled towards each other. Following packing, mechanical ventilation was usually performed. This was typically pressure support ventilation or controlled mandatory ventilation with deep sedation. A transfusion protocol was used including packed red cells, fresh frozen plasma and concentrated platelets. No topical haemostatic or systemic procoagulant agents (eg cryoprecipitate, fibrinogen or recombinant factor VIIa) were used.

**Figure 1:**
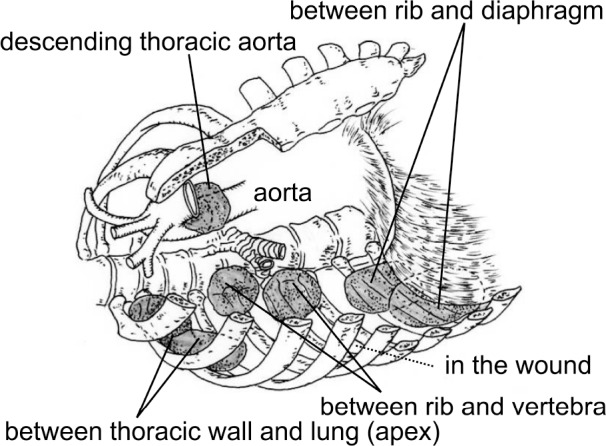
Schema of the packing site in the thoracic cavities of the six successful cases

## Results

Seven of the twelve cases (58%) achieved successful haemostasis for thoracic bleeding after the sequence of DC procedures. Six cases (50%) underwent depacking and definitive surgery for the thorax. Five patients (42%) survived to discharge. One died of retroperitoneal and pelvic trauma, and one died of multiorgan dysfunction after successful haemostasis for thoracic bleeding. The cause of injury was a traffic accident in five patients, a free fall in five patients, a gunshot wound in one patient and being struck by a falling object in one patient. All five cases injured by a free fall died. One of these had achieved successful haemostasis for thoracic bleeding.

Four of the twelve patients underwent a simultaneous resuscitative laparotomy. Two of these survived to discharge but the other two did not despite achieving successful haemostasis for thoracic bleeding.

Five of the twelve patients underwent selective angiography and transcatheter embolisation prior to the initial DC procedure, and two of these underwent embolisation for the chest (intercostal arteries and bronchial artery). Four of the five patients died.

The major origins of haemorrhage in the thoracic cavity included the thoracic wall in seven cases, the lung in eight cases, the vertebrae in one case and the descending aorta in one case. Nine patients displayed injured extrathoracic parts (the abdomen in six, the pelvis in three, the lower extremities in six and the head in one). Five of these (56%) survived to discharge. Five patients fell into cardiac arrest during DC surgery. Four of these died during the procedure and one survived to discharge (Table 1).

The mean values of prothrombin time, active thrombin time, base deficit and body temperature at the time of arrival at the emergency department were 49 seconds, 122 seconds, 13.1mEq/l and 35.5ºC for intrathoracic haemorrhagic cases that were controlled. The corresponding values for the non-controlled intrathoracic haemorrhagic cases were 62 seconds, 239 seconds, 15.1mEq/l and 36.0ºC respectively. There was no statistical difference for any of the parameters between these groups.

Gauze packing was performed for the thoracic wall in five patients, for the lung in four patients, for the vertebrae in two patients and for the descending thoracic aorta in one patient. Three of the thoracic wall patients (60%), one of the lung patients (25%), one of the vertebrae patients (50%) and one of the descending thoracic aorta patients achieved successful haemostasis for thoracic bleeding (Fig 1).

**Figure 2:**
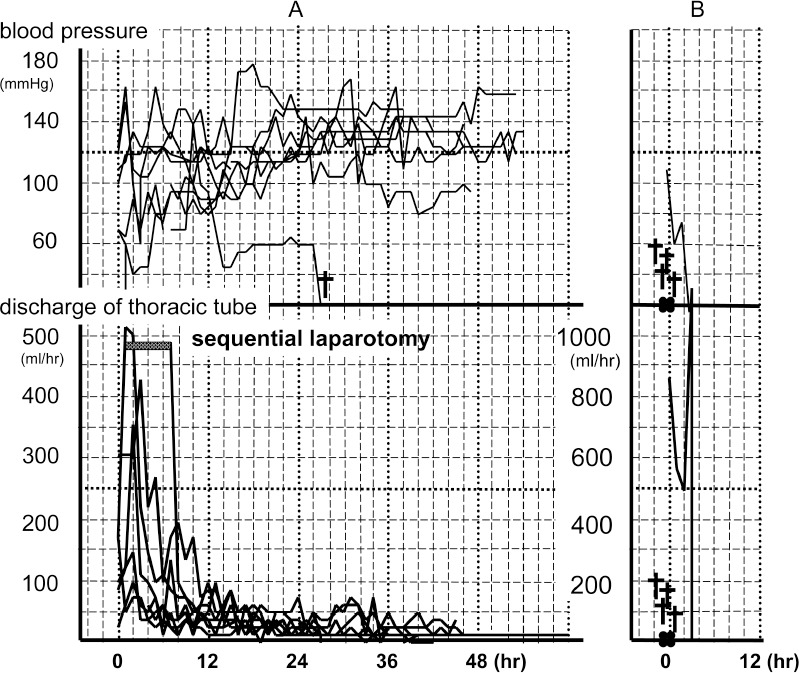
Time course for the systolic blood pressure and volume of discharge from the thoracic tube of patients whose haemorrhage could be stopped by gauze packing (A) and those in whom haemorrhage could not be stopped (B)

Packing for the thoracic aorta, around the apex, diaphragm and vertebrae, as well as in spaces enclosed by the thoracic wall, ribs, vertebrae and diaphragm, tended to result in successful haemostasis. In the five non-controlled intrathoracic haemorrhagic cases, in which the patients died of exsanguination, four patients suffered cardiac arrest during DC surgery and died immediately following the procedure. Although the fifth patient was able to bear the surgery, the volume of discharge from the thoracic drain exceeded 400ml/hr continuously until her death at four hours after surgery. In this patient, the packing sites were the lateral thoracic wall and the lung surface (Fig 2).

**Table 1:** Details of the cases

Case	Age	Mechanism	Survived / died	Site of major bleeding	Site of packing	Extrathoracic injuries	Intrathoracic procedure before packing	Treatment prior to DC	Treatment simultaneous with DC	RBC	FFP
1	19	TA	S	TW (diaphragm), lung	TW (diaphragm), lung	Pelvis, liver	Partial resection of lung		Laparotomy	4	4
2	55	Crush	S*	TW (back)	TW (back)	Retroperitoneal	Suture of TW		Laparotomy	11	3
3	54	Gun shot	S	Vertebrae, TW (back)	Vertebrae, TW (back)		Suture of lung			8	10
4	69	TA	S	TW, lung	TW		Lobectomy			52	66
5	24	Train	S	TW, lung	TW	Pelvis, femoral	Lobectomy		Amputation of the leg	34	20
6	36	TA	D**	Aorta, TW, lung	Aorta	Liver	Replacement of aorta	TAE (liver)	Laparotomy	154	108
7	22	Fall	D**	TW (apex, diaphragm), lung	TW (apex, diaphragm), lung	Spleen, kidney, pelvis	Suture of TW	TAE (pelvis, spleen, kidney)	Laparotomy	44	60
8	4	TA (walking)	D*	Lung (U-M), TW	Lung (U-M)					16	8
9	28	Fall	D*	TW (diaphragm), lung	TW (diaphragm), lung					26	32
10	21	Fall	D*	TW (lateral), lung	TW (lateral), lung			TAE (ICA, BA)		30	32
11	70	Fall	D	Vertebrae, TW (back), lung	Vertebrae, TW (back), lung			TAE (kidney, lumbar)		8	2
12	25	Fall	D*	TW (back), lung	TW (back), lung	Pelvis	Suture of lung	TAE (IC, pelvis)		52	66

TA = traffic accident; S = survived to discharge; D = died; TW = thoracic wall; U = upper lobe; M = middle lobe; DC = damage control;

TAE = transcatheter arterial embolisation; ICA = intercostal artery; BA = bronchial artery; RBC/FFP = units of red blood cells/fresh frozen plasma used before and during damage control surgery

* Cardiac arrest during damage control surgery

** Died after establishing thoracic haemostasis

In the seven patients in whom haemostasis for thoracic bleeding was successful, during thoracotomy and before gauze packing, ligation or electrical coagulation was performed at points in the thoracic wall and on the lung surface that were bleeding. Partial resection of the lung was also carried out using automatic suture devices in three cases, electrical coagulation of the vertebrae in one case, fixation of a fractured rib in one case and replacement of the thoracic aorta in one case.

The volume of the discharge from the thoracic tube exceeded 400ml/hr within 3 hours after packing in 3 cases (43%) and 300ml/hr within this time in 4 cases (57%). Although the volume was >200ml/hr in 3 cases (43%) at 3 hours after packing, discharge decreased to <200ml/hr at hours 4, 5 and 7 for these 3 patients. In the last case, a sequential laparotomy was performed for seven hours after intrathoracic packing. The discharge exceeded 400ml/hr initially and decreased to 200ml/hr after this laparotomy. In 6 cases (86%), the discharge decreased to 100ml/hr within 8 hours. In the remaining case, it decreased to 100ml/hr within 11 hours. We were able to maintain systolic blood pressure at >70mmHg at 7 hours and >100mmHg at 9 hours after packing (Fig 2).

**Figure 3:**
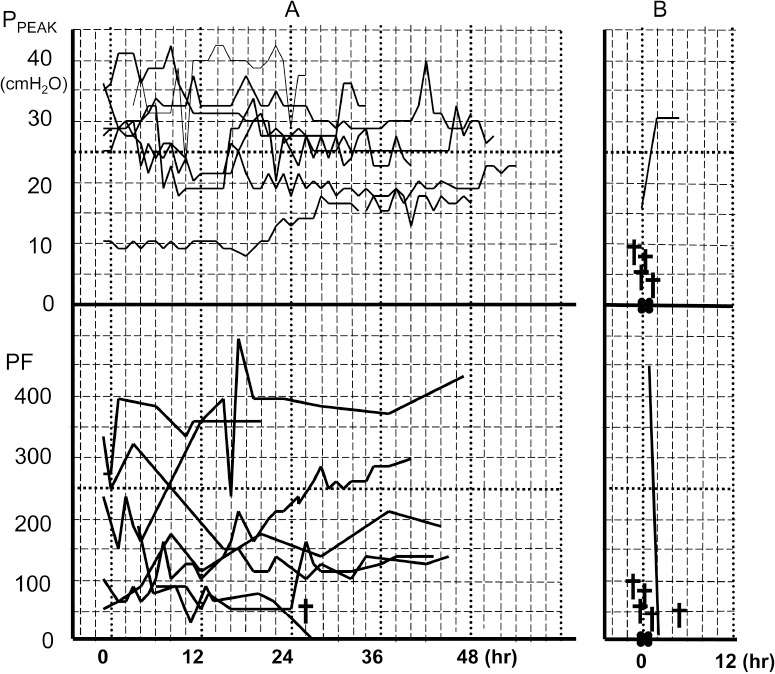
Time course for the peak airway pressure and PaO_2_/ FiO_2_ ratio of patients whose intrathoracic haemorrhage could be stopped by gauze packing (A) and those in whom haemorrhage could not be stopped (B)

The mean duration of packing in the 6 patients who underwent depacking was 52.7 hours (range: 32–88 hours). There were no infectious complications derived from the gauze packing. Concerning respiratory function, oxygenation was generally poor. The patients’ peak airway pressure and P/F ratios are shown in Figure 3. The PaO_2_/FiO_2_ (P/F) ratios of 3 of the patients who achieved successful haemostasis were <150. The P/F ratio of another patient with successful haemostasis was <250 for the first 12 hours after packing. In another such patient the P/F ratio was higher initially but then decreased to <250 for the period of 12–26 hours after packing. The P/F ratio for each of these patients improved gradually and consequently recovered. Airway pressure was generally high. The peak airway pressure of three of the patients achieving successful haemostasis was >25cmH_2_O and that of another such patient was >25cmH_2_O for the first 7 hours after packing.

## Discussion

Gauze packing is one of the most familiar and accepted haemostatic procedures for initial DC surgery, particularly in abdominal trauma.^1–6^ The indications for DC for abdominal trauma are normally haemorrhagic shock due to intraabdominal bleeding (confirmed by abdominal ultrasonography or focused assessment by sonography for trauma) with acidosis, coagulopathy and hypothermia (lethal triad) or a condition worsening progressively towards the lethal triad.^13^ However, we also performed gauze packing as DC surgery in situations where these criteria were absent but where it seemed to be impossible to control the bleeding by any other method, although this resulted in a very poor outcome (survival rate 50%). There are no other studies reporting survival rates from chest DC worthy of evaluation owing to the limited number of relevant cases. However, our data showed we may have treated some cases that were only saved owing to the use of this procedure.

In practical terms, it is difficult to ascertain coagulopathy because in Japan blood tests are generally performed just after the arrival of the patient in the emergency department. At this time, coagulation tests do not usually show lethal abnormalities and we cannot perform these tests repeatedly over a short period of time. Furthermore, we cannot evaluate body temperature repeatedly as the procedure for temperature measurement may disturb the treatment.

Some authors have suggested that the indications for DC are significant massive bleeding (>10 units of red blood cells), coagulopathy (activated partial thromboplastin time >60 seconds), hypothermia (<34°C), an injury severity score of >35, acidosis (pH <7.2) and a prolonged shock phase.^14,15^ In our study, as we could not show lethal conditions with respect to coagulation and body temperature and as we could not show any difference between cases in which haemostasis was successful or not, we have been unable to clarify the indications for DC using the values obtained from the base deficit, temperature and coagulation tests.

In DC gauze packing, with pressure high enough to surpass the highest local circulating pressure and tissue pressure, we leave gauze in a space enclosed by tissue that either does not stretch or only stretches minimally, such as bones, fascia or strong muscles. This technique increases the pressure in the packed gauze. During abdominal packing in DC surgery for severe abdominal trauma, gauze left in the abdominal or retroperitoneal cavity can be compressed by the abdominal wall. However, although the thoracic cavity is enclosed by the costal basket, it is too large to be filled by gauze packing and the bleeding points cannot be compressed by an extracorporeal compressor. Some authors therefore say it is difficult to control massive haemorrhage due to severe thoracic wall injury using intrathoracic gauze packing.^11^

Moreover, increased intrathoracic pressure due to the presence of the bulky gauze as well as the gauze itself induce cardiopulmonary collapse and dysfunction by compression of the right side of the heart, the hilar pulmonary vessels and the superior caval vein. Desaturation and ventilation disorders are also induced by disturbing the expansion of the lung and normal movement of the diaphragm, particularly in patients with severe lung contusion. Nevertheless, there have been no reports concerning the adverse effects of intrathoracic packing for cardiopulmonary function and the threshold of safety for this procedure. We usually hesitate to perform gauze packing during DC surgery. One half of the patients who achieved successful haemostasis in our study suffered from severe respiratory distress, desaturation and high airway pressure after packing but their respiratory conditions improved after haemostasis and depacking.

This study clarified that there are some cases with massive, rapid and uncontrollable intrathoracic haemorrhage induced by simple thoracic drainage in which gauze packing is useful for haemostasis. Unfortunately, because we perform DC surgery/intrathoracic gauze packing for patients who we think will die of exsanguination without this procedure, we were not able to evaluate in this small case series for which cases and types of bleeding this procedure is unnecessary.

Successful haemostasis may be achieved by packing around the apex, diaphragm and vertebrae. The decision to perform this procedure is made in conjunction with other potentially lethal factors affecting thoracic haemorrhage such as extrathoracic bleeding, infection and injuries outside the chest. Nevertheless, if we extend the indications for this procedure to less severe cases, there may be more cases in which intrathoracic packing is potentially useful with respect to the duration of haemostasis, the duration of intensive care unit stay, the extent of mechanical ventilation, the duration of absolute rest and the necessary volume of blood transfusion as well as in terms of cost.

We sought to describe the methodology of gauze packing in cases in which haemostasis was successful and the characteristics of intrathoracic injuries with bleeding. We could also show the pattern and change in the volume of discharge from the thoracic drain and the haemodynamic conditions of cases with successful haemostasis. This can be helpful for surgeons and traumatologists who have to perform intrathoracic packing for the first time postoperatively owing to uncontrolled bleeding.

In cases with uncontrolled bleeding, it is important to decide aggressively and quickly whether to repack or to perform other procedures. However, the results presented here suggest that it is more important to wait and not repack hurriedly in cases in which the volume of discharge from the thoracic drain and the haemodynamic conditions after packing are thought to be acceptable. We recommend waiting for at least three hours after packing, when the vital signs of patients can be maintained with appropriate blood transfusion and when the volume of discharge from the thoracic tube decreases. The physician should wait to determine whether the discharge decreases to <200ml/hr within 4 or 5 hours.

There are few reports concerning the permissible duration of compression by gauze or retention of foreign bodies based on clinical or experimental data.^5,10,16,17^ In an effort to establish successful haemostasis, a longer duration may be better. On the contrary, from the viewpoint of inflammation risk, infection or adverse effects on respiratory and circulatory conditions of shorter duration may be preferable.^2,3,5,13,16–20^

In the present study, the duration of intrathoracic packing ranged from 32 to 88 hours in the cases in which haemostasis was successful and the mean duration was 52.7 hours. The required duration depended not only on the nature of the intrathoracic bleeding but also on the volume and speed of bleeding from other parts of the body as well as other factors. We cannot define a standard duration of intrathoracic packing owing to the limited number of cases for which intrathoracic packing was inevitable. We have previously reported on clinical research concerning the relation between the duration of packing and the development of bacterial cultures in the packed gauze in patients who underwent abdominal DC.^13,17^ We concluded that 96–120 hours is an acceptable duration in terms of the risk of infection. In thoracic DC, it is advisable to remove packed gauze within three or four days.

## Conclusions

Intrathoracic gauze packing is useful as the initial surgery of DC for some patients with massive and rapid thoracic haemorrhage with shock. Intrathoracic packing may be effective in particular locations in the thoracic cavity such as the space enclosed between bones, around vertebrae, at the lung apex, and between the diaphragm and thoracic wall. This procedure should be attempted for lethal thoracic haemorrhagic patients. It is advisable to wait for at least three hours after packing if the vital signs of the patient can be maintained with appropriate blood transfusion. The physician should continue to wait if the volume of the thoracic tube discharge decreases to <200ml/hr within 4 or 5 hours. Packed gauze should be removed within three or four days.

## Retraction of duplicate publication

Recently, we were made aware of a case of duplicate publication.^1,2^ A technical tip published in the *Annals* in 2009 had been published previously in *BMJ Case Reports*. Our enquiries, conducted jointly with *BMJ Case Reports*, established that the corresponding author had submitted the same material to the *Annals* immediately after it had been accepted for online publication in *BMJ Case Reports*. The text and authorship of the two publications are identical. Duplicate publication of any sort is publication malpractice, which wastes resources, including the time of editors and reviewers, as well as publication space that could be offered to other, original work. Both journals will deal robustly with any confirmed example.

This article was submitted as a duplicate publication to *BMJ Case Reports* and the *Annals* in 2008. On discovery of this in 2012, following the indexing of *BMJ Case Reports*, the article in the *Annals* has been retracted as per the Committee on Publication Ethics guidelines. Manuscripts may not be duplicated. This includes submissions to online as well as paper journals, regardless of indexing. Authors unsure as to whether parts of manuscripts or different aspects of their research may be submitted to multiple journals are advised to contact the editorial teams of the respective journals before submission, to include this in their disclosures and to check carefully the final drafts of the manuscripts before publication online or on paper. Further advice is available at http://publicationethics.org/.
